# Analyzing the Release of Copeptin from the Heart in Acute Myocardial Infarction Using a Transcoronary Gradient Model

**DOI:** 10.1038/srep20812

**Published:** 2016-02-11

**Authors:** Jes-Niels Boeckel, Jana Oppermann, Remzi Anadol, Stephan Fichtlscherer, Andreas M. Zeiher, Till Keller

**Affiliations:** 1 Department of Cardiology, Internal Medicine III, Goethe-University Hospital, Theodor Stern Kai 7 60590 Frankfurt; Germany; 2 Institute for Cardiovascular Regeneration, Center of Molecular Medicine, Theodor Stern Kai 7, 60590 Frankfurt; Germany; 3 German Center of Cardiovascular Research (DZHK), RheinMain, 60590 Frankfurt, Germany

## Abstract

Copeptin is the C-terminal end of pre-provasopressin released equimolar to vasopressin into circulation and recently discussed as promising cardiovascular biomarker amendatory to established markers such as troponins. Vasopressin is a cytokine synthesized in the hypothalamus. A direct release of copeptin from the heart into the circulation is implied by data from a rat model showing a cardiac origin in hearts put under cardiovascular wall stress. Therefore, evaluation of a potential release of copeptin from the human heart in acute myocardial infarction (AMI) has been done.

Copeptin is the small C-terminal portion of the antidiuretic pre-proprotein of arginine vasopressin (proAVP). AVP is generally released in response to stress on the circulatory system such as an increase in plasma osmolality resulting in an antidiuretic effect^1^. Copeptin is released in the same amount as AVP. However AVP has only a half-life of 5–20 min in plasma compared to days for copeptin^2^,^3^. Therefore, copeptin has been established as a reliable biomarker for heart diseases as well as a predictor of mortality instead of AVP^4^,^5^,^6^.

Elevation of AVP and copeptin can be observed in a variety of pathophysiological conditions e.g. type 2 diabetes^7^,^8^, pneumonia^9^, acute pancreatitis^10^,^11^, sepsis^12^,^13^ but also cardiac stress and injury^14^,^15^,^16^,^17^. Besides the established biomarkers for cardiac injury like cardiac troponins, copeptin levels might provide additional information regarding circulatory stress levels and hemodynamic instability. Therefore, copeptin has shown to provide amendatory diagnostic information for early discrimination of e.g. acute myocardial infraction (AMI) in combination with cardiac troponins in most published studies^14^,^15^,^16^,^17^.

AVP is usually synthesized in the hypothalamic supraoptic (SON) and paraventricular (PVN) nuclei and released from the neurohypophysis into the circulation^18^. In one published study^19^ isolated rat hearts were put under elevated cardiovascular wall stress which led to increased levels of vasopressin on mRNA and on peptide level. Therefore, this study implies a potential release of copeptin from the heart into the circulation. Since then, evidence for a release of Vasopressin and copeptin has only been show from the hypophysis, if the heart also contributes to a release into the blood is matter of an ongoing debate.

Therefore, we analyzed a potential release from the heart by measuring the concentration of copeptin using a transcoronary gradient model (TCG) in patients with AMI.

## Methods

### Study cohort

Patients who underwent a coronary angiography at the University Hospital Frankfurt between October 2009 and September 2010 were enrolled as described earlier in detail^20^. In brief, they were classified as AMI patients in case of presence of a relevant coronary artery disease in the performed angiography and elevated cardiac troponin T (cTnT) levels and as non-AMI in case of low cTnT levels. Pre-defined exclusion criteria were known history of leukopenia, thrombocytopenia, severe hepatic disorder, severe renal dysfunction, sepsis, pancreatitis, ongoing inflammatory or malignant disease and the diagnosis of myocarditis or cardiogenic shock at presentation. The local ethics review board of the Goethe University Frankfurt (Frankfurt, Germany) approved the protocols, and the study was conducted in accordance with the Declaration of Helsinki. Written informed consent was obtained from each individual.

### Sample collection and laboratory methods

Blood was simultaneously collected from the aortic bulb (AO) and the coronary venous sinus (CVS) during a standard cardiac catheterization procedure before heparin or any contrast agent was administered and before any interventional procedure was started as described previously^20^. The TCGs for individual biomarkers were calculated by subtracting the AO from the CVS levels.

After centrifugation, plasma and serum samples were transferred to RNase/DNase-free tubes and stored at −80 °C within 1–2 hours.

Copeptin levels were measured in CVS and AO blood samples in 50 μl serum using the copeptin US assay on a KRYPTOR compact PLUS (BRAHMS Thermo Scientific) in accordance with the manufactures instructions. Measurements were carried out by experienced staff blinded to patient characteristics.

CTnT was measured using a commercially available highly sensitive assay (Roche diagnostics) at the central laboratory of the recruiting institution. As no established 99^th^ percentile cut-off is available for cTnT measurements using CVS blood, a concentration of 100 pg/ml was used as discriminatory threshold.

### Statistics

The p-values in the baseline table refer to the exact Fisher’s test, t- and Wilcoxon tests for categorical, symmetric and skewed distributions, respectively. A possible association between chest pain onset time, and copeptin TCG levels were analyzed by calculation of the Spearman correlation coefficients. All analyses were carried out using the R software package version 3.1 (R Foundation for Statistical Computing, Vienna, Austria).

## Results

To determine whether the heart contributes to the release of copeptin into the bloodstream in humans, we measured copeptin levels in patients suffering an AMI and in patients without AMI. A detailed characteristic of the study cohort is provided in table 1.

Levels of cTnT, as established biomarker representing myocardial ischemia, as well as copeptin were determined in samples from the aorta (AO) and the coronary venous sinus (CVS) in patients with (n = 15) and without (n = 14) the diagnosis AMI. Of those, 5 suffered a non-ST elevation myocardial infarction whereas 10 showed significant ST elevation in the electrocardiogram.

### Transcoronary release of cardiac troponin T in acute myocardial infarction

**Figure 1:**
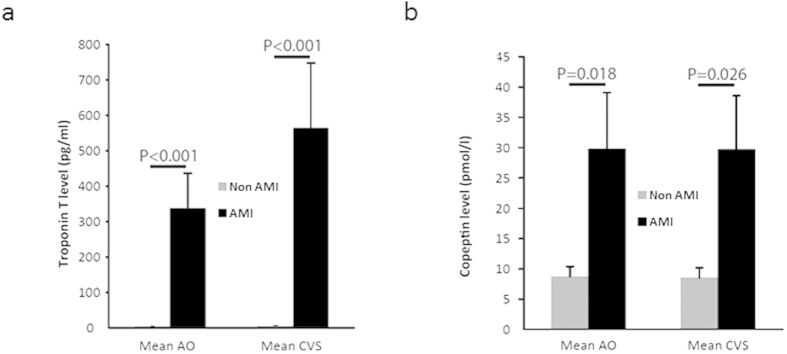
(**a**) Level of aortic and CVS cardiac troponin T, (**b**) copeptin levels in patients with acute myocardial infarction (AMI; n = 15) compared to patients without AMI (non-AMI; n = 14).

**Figure 2:**
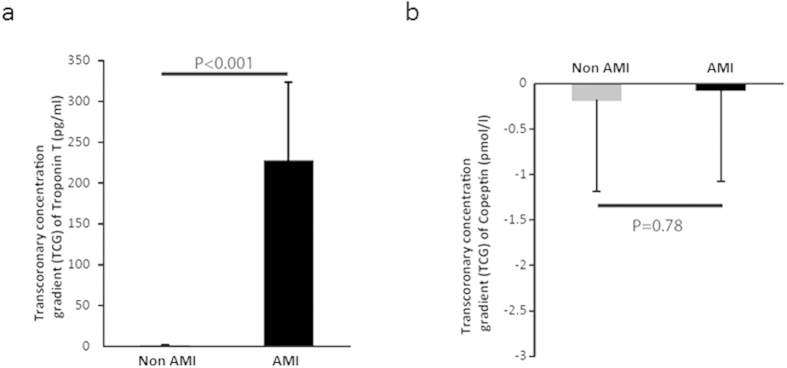
(**a**) Level of transcoronary cardiac troponin T and (**b**) copeptin gradients in patients with acute myocardial infarction (AMI; n = 15) compared to patients without AMI (non-AMI; n = 14). Data is shown as mean ± SEM. Statistical differences between groups were analysed using the Wilcoxon test.

In the patient group without an AMI we found a mean cTnT concentration of 4.15 pg/ml (±0.66) in AO samples, while 5.59 pg/ml (±0.74) was detected in the CVS (Fig. 1A). This resulted in a slightly positive transcoronary gradient (TCG) concentration for cTnT of 1.45 pg/ml (±0.58) (Fig. 2A). In contrast, in AMI patients we found a significantly (P < 0.001) higher cTnT AO concentrations of 337.17 pg/ml (±98.95) and of 564.47 pg/ml (±183.14) in the CVS (P < 0.001) (Fig. 1A). This resulted in a relevant positive higher TCG of 227.30 pg/ml (±96.34) (Fig. 2A) compared to non-AMI patients (P < 0.001). If restricting these analyses to AMI patients without ST elevations a comparable positive TCG of 76.10 pg/ml (±58.61) was observed.

### Transcoronary gradient of copeptin

In the non-AMI group we found mean AO copeptin levels of 8.73 pmol/l (±1.67) and of 8.54 pmol/l (±1.64) in the CVS (Fig. 1B). In line with recent publications^14^,^17^, we could detect significantly (P = 0.018) increased levels of copeptin in AMI patients measured in the AO 29.78 pmol/l (±9.32) as well as a significantly (P = 0.026) increased levels in the CVS with 29.70 pmol/l (±8.85) (Fig. 1B). This difference between AMI and non-AMI patients leads to a diagnostic information of copeptin quantified by the area under the curve in the receiver operator characteristics analyses of 0.74 (0.55–0.93) for copeptin measured in the CVS and of 0.76 (0.57–0.94) if determined in AO blood.

However, no relevant release or uptake of copeptin during the transcoronary blood passage could be detected when comparing the mean TCG of patients with or without the diagnosis AMI with −0.07 and −0.18 pmol/l (P = 0.78), respectively (Fig. 2B). Considering only AMI patients without ST elevations likewise no relevant cardiac copeptin release could be observed with a TCG of 0.02 pg/ml (±0.29).

Additionally, the potential association of a transcoronary copeptin gradient and time between symptom onset and time of blood sampling was evaluated. This time interval did not show a relevant correlation with the TCG of copeptin (correlation coefficient: –0.072; p = 0.80). Furthermore, AMI patients presenting early, within 4 hours after onset of symptoms (n = 7), showed no difference in TCG of copeptin with mean of –0.185 pmol/l compared to 0.021 pmol/l (p = 0.91) as determined in patients presenting later than 4 h.

## Discussion

Timely diagnosis and especially a valid early rule-out of an AMI are crucial already in the emergency department. Amendatory to the established biomarkers, which are released mainly from the heart due to myocardial injury, like cardiac troponins, copeptin has shown diagnostic value. Copeptin is increased by several disease processes besides the cardiovascular system and is therefore a more universal biomarker for severe patients’ physiology^21^. Within the recent years, several publications demonstrated that copeptin indeed provides additional information for risk stratification and rule-out of AMI when combined with cardiac troponins^22^,^23^.

Aside from the fact that AVP and copeptin are usually synthesized in the hypothalamic supraoptic (SON) and paraventricular (PVN) nuclei and released from the neurohypophysis into the circulation^18^, the potential release from the heart has been raised by evidence of AVP mRNA and protein expression in rat hearts put under elevated cardiovascular wall stress^19^.

Within the present study, we measured copeptin levels before the transcoronary blood passage in the AO and after the heart in the CVS draining the myocardium. In the patients that did not suffer an AMI we determined copeptin with a mean 8.72 pmol/l (±1.66) in blood taken form the AO and with 8.54 pmol/l (±1.64) in CVS blood resembling non-diseased levels in the same magnitude as published in healthy individuals found with median of 4.3 pmol/l and of 3.2 pmol/l in females and males respectively^24^ and with published upper 95^th^ percentile reference limit of 9.8 pmol/l as potential diagnostic threshold in evaluation of suspected AMI^17^. Regarding the potential cardiac release of copeptin, we did not observe a positive TCG gradient suggesting a non-cardiac origin of copeptin.

In line with several recent publications we demonstrate a clear increase of copeptin in patients with AMI^14^,^17^,^25^ well above the mentioned cut-off with significant higher levels of copeptin in the aorta and the CVS of AMI patients compared to those without AMI that’s does not originate directly from the myocardium. As expected, we saw a profound positive and significant TCG for cTnT in samples from AMI patients indicating a progressive release from the heart muscle, in contrast to copeptin levels before and after the heart remained unchanged.

However, copeptin and cTnT have different release kinetics after onset of chest pain. Patients with myocardial infarction might even have highest copeptin levels already at the time of admission to the emergency department^14^,^17^. Therefore, the levels of copeptin in our study might already been equalized by saturation of the aortic and venous system. In such case, a release of copeptin resulting in a positive TCG would no longer be detectable when comparing AO and CVS copeptin levels. Given that the median time since onset of chest pain in the present cohort was 5 h (25^th^ percentile 2.5 h, 75^th^ percentile 9 h), quite comparable to the published results, and furthermore no relevant association of copeptin TCG with chest pain onset time was observed in our cohort, this seems not very likely. Moreover, in a human model for myocardial infarction in patients undergoing transcoronary ablation of septal hypertrophy, a significant increase of copeptin was seen as early as 30 min after induction of myocardial infarction lasting for 240–480 min^26^ putting our evaluated patients in the relevant timeframe.

One has to acknowledge the aspect, that we measured copeptin as surrogate marker for AVP. Still, given the assumed equimolar release of AVP and copeptin and the preferable preanalytics of copeptin this should not limit the general information on transcoronary release as reported.

To conclude, in line with previous reports we found a significant increase of copeptin in patients suffering an AMI supporting the use as diagnostic biomarker. Using a TCG model we could not detect a relevant direct cardiac release of copeptin into the coronary circulation in AMI patients. In summary even if there is an expression of AVP and copeptin in the human heart, the present study indicates that a direct release of copeptin is not detectable from the human heart in acute myocardial infarction.

## Additional Information

**How to cite this article**: Boeckel, J.-N. *et al.* Analyzing the Release of Copeptin from the Heart in Acute Myocardial Infarction Using a Transcoronary Gradient Model. *Sci. Rep.* **6**, 20812; doi: 10.1038/srep20812 (2016).

## Figures and Tables

**Table 1:** Baseline characteristics of the study cohort (n = 29).

	Number of patients	AMI	Number of patients	non- AMI	p-value
Male gender	15	12/15 (80%)	14	13/14 (93%)	0.598
Age (years), mean (SD)	15	62.1 (10.9)	14	56.7 (9.8)	0.174
NSTEMI		5/15 (33%)			
STEMI		10/15(66%)			
Cardiovascular risk factors					
Hypertension	15	10/15 (67%)	14	10/14 (71%)	1
Hyperlipidemia	14	1/14 (7%)	14	1/14 (7%)	1
Diabetes mellitus	15	4/15 (27%)	14	5/14 (36%)	0.561
Active smoker	14	6/14 (43%)	14	8/14 (57%)	0.705
Obesity	10	4/10 (40%)	14	7/14 (50%)	0.697
Family history	13	1/13 (8%)	13	1/13 (8%)	1
Ejection fraction	15/12	44 ± 10	14/12	51 ± 13	0.19
Laboratory parameters on admission					
Troponin T - CVS (pg/mL)	15	564.47 ± 183.14	14	5.59 ± 0.74	*<0.001*
Troponin T - AO (pg/mL)	15	337.17 ± 98.95	14	4.15 ± 0.66	*<0.001*
Troponin T - TCG (pg/mL)	15	227.2 ± 96.3	14	1.45 ± 0.58	*<0.001*
Copeptin - CVS (pmol/L)	15	29.70 ± 8.85	14	8.54 ± 1.64	*0.018*
Copeptin - AO (pmol/L)	15	29.78 ± 9.32	14	8.73 ± 1.67	*0.026*
Copeptin - TCG (pmol/L)	15	−0.07 ± −0.86	14	−0.18 ± −0.32	*0.78*
NT-proBNP - CVS (pg/mL)	15	959.04 ± 272.45	14	120.20 ± 23.63	*0.02*
NT-proBNP - AO (pg/mL)	15	767.95 ± 233,76	14	90.27 ± 23.50	*0.014*
NT-proBNP - TCG (pg/mL)	15	191.09 ± 80.74	14	29.92 ± 7.81	*0.354*
Clinical variables					
Chest pain onset time [h], median (IQR)	15	5.0 (2.5, 9)			

Data are presented as n (%), mean (standard error of the mean) or median (25th, 75th percentile). AMI denotes Acute Coronary Syndrome, NSTEMI denotes Non ST-elevation myocardial infarction, STEMI denotes STelevation myocardial infarction. Obesity was defined as body mass index of at least 30. Data is shown as percentage, mean (SEM) for troponin and copeptin and median (IQR) for chest onset time. P-values in the baseline table refer to chi square, t- and Wilcoxon tests for categorical, and continuous symmetric and skewed distributed variables, respectively.
